# Genomic Analysis of Resistance to Fall Armyworm (*Spodoptera frugiperda*) in CIMMYT Maize Lines

**DOI:** 10.3390/genes13020251

**Published:** 2022-01-28

**Authors:** Isaac Kamweru, Bruce Y. Anani, Yoseph Beyene, Dan Makumbi, Victor O. Adetimirin, Boddupalli M. Prasanna, Manje Gowda

**Affiliations:** 1 International Maize and Wheat Improvement Center (CIMMYT), Village Market-00621, Nairobi P.O. Box 1041, Kenya; ink@udel.edu (I.K.); A.Bruce@cgiar.org (B.Y.A.); y.beyene@cgiar.org (Y.B.); d.makumbi@cgiar.org (D.M.); b.m.prasanna@cgiar.org (B.M.P.); 2 Life and Earth Sciences Institute (Including Health and Agriculture), Pan African University, Ibadan 200284, Nigeria; 3 Department of Agronomy, University of Ibadan, Ibadan 200284, Nigeria; vo.adetimirin@ui.edu.ng

**Keywords:** maize, fall armyworm, genomic analysis, native genetic resistance, single nucleotide polymorphism

## Abstract

The recent invasion, rapid spread, and widescale destruction of the maize crop by the fall armyworm (FAW; *Spodoptera frugiperda* (J.E. Smith)) is likely to worsen the food insecurity situation in Africa. In the present study, a set of 424 maize lines were screened for their responses to FAW under artificial infestation to dissect the genetic basis of resistance. All lines were evaluated for two seasons under screen houses and genotyped with the DArTseq platform. Foliar damage was rated on a scale of 1 (highly resistant) to 9 (highly susceptible) and scored at 7, 14, and 21 days after artificial infestation. Analyses of variance revealed significant genotypic and genotype by environment interaction variances for all traits. Heritability estimates for leaf damage scores were moderately high and ranged from 0.38 to 0.58. Grain yield was negatively correlated with a high magnitude to foliar damage scores, ear rot, and ear damage traits. The genome-wide association study (GWAS) revealed 56 significant marker–trait associations and the predicted functions of the putative candidate genes varied from a defense response to several genes of unknown function. Overall, the study revealed that native genetic resistance to FAW is quantitative in nature and is controlled by many loci with minor effects.

## 1. Introduction

Maize (Zea mays L.) is a staple food for over 300 million people on the African continent [1]. To achieve maize-based food security in the region by 2050, the current average maize production of about 1.5 tons/ha has to increase to 6.8 tons/ha [2]. In the context of the present and forecasted climatic patterns, overall maize production in the next three decades has been projected to reduce by about 10% [3]. The fall armyworm (FAW) destructive pest, first reported in 2016 in Africa [4] but now confirmed in 46 of Africa’s 54 countries, is likely to establish itself as a multi-generational pest of economic importance in Africa due to its natural distribution capacity, high fecundity level, favorable sub-tropical climates, wide host range, voracious appetite, and migratory activities [5,6].

FAW foliar feeding, especially on the furl and whorl leaves, destroys the plant growing points and retards maize growth and development. Yield reduction has been attributed to both FAW stem tunneling, which disrupts water and nutrient uptake, as well as the extensive leaf feeding damage which causes a direct loss of photosynthates [7]. FAW-damaged ears are also predisposed to fungal attacks, rots, and mycotoxin contamination, which adversely impact grain quantity and quality [8]. Globally, about 6–19% of the total maize production is lost to insect-pests herbivory [9]. Failure to manage the FAW infestation in sub-Saharan Africa is projected to result in an annual loss of approximately 12% of the total area under maize cultivation (~37 million hectares), which translates to economic losses of up to US $6.3 billion per annum [6]. A wide distribution of direct and indirect maize yield losses has been reported throughout the FAW’s native and invasive range [10]. In sub-Saharan Africa for instance, annual losses of up to US$ 13 billion have been estimated in crops such as maize, rice, sorghum, and sugarcane [11,12].

Transgenic Bt crops provide an effective FAW control but impose a high selection pressure which results in the emergence of resistant FAW biotypes [13,14], while the application of synthetic pesticides in FAW control raises environmental toxicity concerns [15] and could also be ineffective in the future due to the emergence of resistant FAW biotypes [16]. The majority of African farmers facing maize yield losses due to FAW infestation are resource-poor and have limited local access to partially effective chemical and biological FAW control measures [17]. These farmers extensively rely on their ecological knowledge to manipulate plant ecology in favor of a few FAW natural predators [18]. The high density of pest populations also renders the eco-friendly, cultural FAW control practices ineffective [19]. The need to protect the maize crop from FAW foliar damage and mitigate yield losses using a multi-pronged strategy that fosters agricultural sustainability is of outmost priority. Cultivating maize varieties endowed with inherent native resistance to foliar damage is not only compatible with biological, chemical, and cultural insect-pest control methods, but is also ecologically and economically sustainable [20].

Host plant resistance is conferred by the plant’s biochemical constituents [21], structural features [22], and genetic composition [23]. The breeding efficiency for trait improvement has increased significantly with the integration of genomic tools such as genomic-wide association studies (GWAS) [24], linkage mapping [25], and genomic selection (GS) [26] with traditional breeding approaches. Genome-wide signals associated with resistance to major lepidopteran pests of maize such as the European corn borer [27], Asian corn borer [28], Southwestern corn borer [29], Sugarcane borer [30], and FAW [31,32] have been detected with improved accuracy and speed in light of the current advances in plant molecular biology, high throughput sequencing technology, and the development of robust statistical data analysis tools. GWAS has received tremendous attention as a quick new alternative for directly scanning diverse sets of maize germplasm for functional polymorphisms at the sequence level [33]. For selecting complex traits like FAW resistance, GS is a promising option. GS can accomplish this by employing genome-wide dense markers for predictions, and therefore can support association analyses to determine the genetic basis of key traits [34].

In the African context, FAW resistance breeding is less well studied [32]. The utilization of African-adapted tropical maize lines developed by CIMMYT could speed up FAW resistance breeding and provide a framework for efficiently pyramiding multiple beneficial alleles [35] in elite but susceptible genotypes. Thus, the objectives of the present study were to (i) assess the genetic architecture of FAW resistance traits such as foliar damage ear damage and grain yield; (ii) identify the significant quantitative trait nucleotides (QTNs) and putative candidate genes for FAW resistance traits in tropical maize germplasm; and (iii) assess the potential of utilizing GS in the improvement of FAW resistance traits. Results from this study could provide baseline information in the genomic-assisted development and release of FAW-resistant maize, which promotes prospects of a better farmer harvest.

## 2. Materials and Methods

### 2.1. Plant Material and Experimental Design

A set of 424 DH lines developed by CIMMYT and 11 elite lines were evaluated for their responses to FAW under artificial infestation. All lines were screened for two seasons in screen houses at the Kiboko experimental station (2°15’S and 37°75’ E, 975 m asl) in Kenya in 2020 and 2021. To control random variation, the experiment utilized an α lattice design with two replications. Test plots consisted of single rows 3 m long and 0.75 m apart. Each year by location combination was considered as a separate test environment. Standard agronomic management practices were implemented up to physiological maturity when the ears were harvested.

### 2.2. Artificial Infestation and Phenotypic Evaluation

Mass FAW rearing was conducted at the insectary within the Kenya Agricultural and Livestock Research Organization, Katumani Agricultural Experimental Station in Machakos county, Kenya using a nutritionally adequate CIMMYT artificial diet [36] under ambient laboratory conditions (temperature of 25 ± 1°C, 12 h day and 12 h dark photoperiod, and relative humidity of 75 ± 5%). Neonate larvae emerged 24 h after eggs hatching. Trial planting dates were adjusted to ensure the desired plant growth stage coincided with peak periods of larvae emergence at the insectary. Eight first instar FAW larvae were manually applied at the furl and whorl leaves of each plant using a camel brush at the V3 stage of maize development [37]. Since leaf tissues are soft at the V3 maize growth stage, it suits the larvae to have them conditioned to the host environment to feed and survive. Fewer FAW larvae have been reported to survive when maize plants were infested at the 12-leaf stage of growth compared to the 8-leaf stage [38]. As the plant growth progresses, leaf tissues become more fibrous and difficult for the larvae to feed on. Previous studies have demonstrated the level of damage sustained by both resistant and susceptible genotypes infested with FAW decline as plants mature [39,40,41]. Therefore, the plants were chosen to be infested with FAW larvae at the V3 stage.

All plots were infested on the same day to ensure the uniformity of infestation. The level of leaf feeding damage for each plant per plot was rated 7, 14, and 21 days after artificial infestation using a visual rating scale of 1–9 [42]. On this scale, 1 = no visible damage, 2 = a few short holes on several leaves, 3 = short holes on several leaves, 4 = several leaves with short holes and a few long lesions, 5 = several holes with long lesions, 6 = several leaves with lesions < 2.5 cm, 7 = long lesions common on one-half of the leaves, 8 = long lesions common on one-half to two-thirds of leaves, and 9 = severe damage, most leaves with long lesions, and complete defoliation. After harvesting, rotten ears were counted per plot and the data were expressed in percentages. FAW-damaged maize kernels are predisposed to infections by *Aspergillus flavus*; as a result, rots develop on individual kernels or part of the ear and result in rotten ears. Grain yield was obtained from the shelled grain weight adjusted to 15% and converted to tons per hectare. Ear damage was rated on a scale of 1–9, where 1 = no visible damage to the ear, 2 = damage to a few kernels ( < 5) or less than 5% damage to an ear, 3 = damage to a few kernels (6–15) or less than 10% damage to an ear, 4 = damage to (16–30) kernels or less than 15% damage to an ear, 5 = damage to (31–50) kernels or less than 25% damage to an ear, 6 = damage to (51–75) kernels or more than 35% but less than 50% damage to an ear, 7 = damage to (76–100) kernels or more than 50% but less than 60% damage to an ear, 8 = damage to >100 kernels or more than 60% but less than 100% damage to an ear, 9 = almost 100% damage to an ear. Considering each year by location as a separate test environment, phenotypic data were taken in two environments with two replications per environment.

### 2.3. Phenotypic and Genotypic Data Analyses

Data from each trait met all the assumptions of the applied statistical model, i.e., normally distributed, constant variance, and independent [43]; as a result, no data transformation was applied. Analyses of variance (ANOVA) was performed for each and across environments, and the restricted maximum likelihood (REML) approach was used in the META-R program [44]. Variance components were calculated with a linear mixed model. The following linear mixed-effects model was used to estimate the variance components in a single environment:

$$Y_{ijk}=\mu +Block_{j}\left(Rep_{i}\right)+Gen_{k}+Cov+e_{ijk}$$

where $Y_{ijk}$ is the trait of interest, µ is the grand mean of the trait, $Rep_{i}$ is the effect of the *i*th replicate, $Block_{j}\left(Rep_{i}\right)$ is the effect of the *j*th incomplete block within the *i*th replicate, $Gen_{k}$ is the effect of the *k*th genotype, Cov is the covariate, and $e_{ijk}$ is the error term associated with the *i*th replication, *j*th incomplete block, and the *k*th genotype. Blocks were considered as random effects while the replicates and genotypes were considered as fixed effects. 

New terms were added to the model when performing trait analysis cross environments. The new linear model used was as follows;

$$Y_{ijkl}=\mu +Env_{i}+Rep_{j}\left(Env_{i}\right)+Block_{k}\left(Env_{i}Rep_{j}\right)+Gen_{l}+Env_{i\;}x\;Gen_{l}+Cov+e_{ijkl}\;$$

where the new terms $Env_{i}$ and $Env_{i\;}x\;Gen_{l}$ are the effect of the *i*th environment and the environment by genotype interaction, respectively. The study treated replication as a fixed effect and all other treatments as random effects. On an entry-mean basis, the broad-sense heritability was estimated using the genotypic to phenotypic variance ratio from the derived variance components. Furthermore, to determine the genotypic effects of the investigated lines for each and across environments, a best linear unbiased estimation (BLUE) and best linear unbiased prediction (BLUP) were obtained.

For GWAS, BLUPs were used. On the other hand, BLUEs were used for GS analyses. Comparisons of variability between entries were made using the least squared differences (LSD) at a 5% significance level. The performance analytics R package [45] was used to compute pairwise Pearson’s correlation coefficients among BLUPs of the phenotypic traits.

Maize leaf tissue samples were collected from eight young, healthy seedlings raised under screen house conditions at the V3 stage (3–4 weeks old). A composite sample of tissues collected from each line was stored at −80 °C and later freeze dried for 72 h. High quality genomic DNA was isolated from freeze-dried tissues using the standard CIMMYT laboratory protocol [46]. The Diversity Array Technology (DArT) marker platform was used to develop 12,906 SNPs. Trait analysis by aSSociation Evolution and Linkage (TaSSEL) [47] was used to summarize genotype data by site, determine the allele frequencies, and implement quality screening. SNP variants that were monomorphic, called at repeat loci, had a heterozygosity of >0.05, and had a minor allele frequency of <0.05 were filtered and 7950 high-quality SNPs were retained for downstream genomic analysis.

### 2.4. Population Structure, Kinship, Linkage Disequilibrium, and GWAS

Principal component analysis (PCA) was implemented using 7950 markers that were distributed across the ten maize chromosomes. A two-dimensional plot of the first two principal components was generated using the multi-locus random SNP effect mixed linear model ‘mrMLM.GUI’ R package version 4.0.2 [48]. Marker-based kinship analysis was used to account for cryptic relatedness in the association mapping panel. A genomic relationship matrix was constructed using a restricted maximum-likelihood (REML) estimate of probability of two alleles at a locus being identical in state [49]. Estimates of linkage disequilibrium (LD) decay over genetic distances were determined by plotting the squared correlation coefficients (r2) between pairs of SNPs against their pairwise physical distance in base pairs using TaSSEL version 5.2 [50]. A non-linear regression model was fitted in the R environment [51] and a population specific critical value of r^2^ = 0.1 was taken, beyond which LD could be due to linkage.

Kinship and population structure were incorporated as covariates in the association analyses. The R package ‘FarmCPU-fixed and random model Circulating Probability Unification’ with GAPIT (Genome Association and Prediction Integrated Tool) was used for GWAS analysis [52]. To evaluate the suitability of the GWAS models used, the relationships between the observed and expected theoretical uniform distribution of *p*-values across all evaluated SNPs were inspected using the diagnostic quantile–quantile plots (Q–Q plots) generated in the R environment using the ‘qqman’ R package [53]. On the Q–Q plots, negative logarithms of the *p*-values from the model fitted in GWAS were plotted against their expected values under the null hypothesis of no association. To summarize GWAS results per chromosome, Manhattan scatter plots were generated by plotting the genomic positions of the SNPs against their negative log base 10 of the *p*-values obtained from the GWAS model, and an F-test was conducted for the null hypothesis on the Y-axis. The source sequences of the significantly associated SNPs were used to perform BLAST searches against the B73 maize reference genome version 3 with a view to identify candidate genes located within a 10 kb window. The inferred biological functions of the candidate genes were retrieved from the maize genome database [54] and published literature.

BLUEs across environments for each trait were used in the GS analysis. The ridge-regression BLUP (RR-BLUP) [55] with a fivefold cross-validation for each trait was used for the analysis. A sample of 6400 SNPs with all data values, equally distributed throughout the genome, and MAF > 0.05 were chosen from the DART data. The GWAS panel was sampled to form a training and prediction set. For each trait, 100 iterations were done for the sampling of the training and validation sets. The prediction accuracy was calculated as the correlation between the observed phenotypes and genomic estimated breeding values (GEBVs) divided by the square root of heritability [56].

## 3. Results

### 3.1. Phenotypic Data Analyses

**Figure 1:**
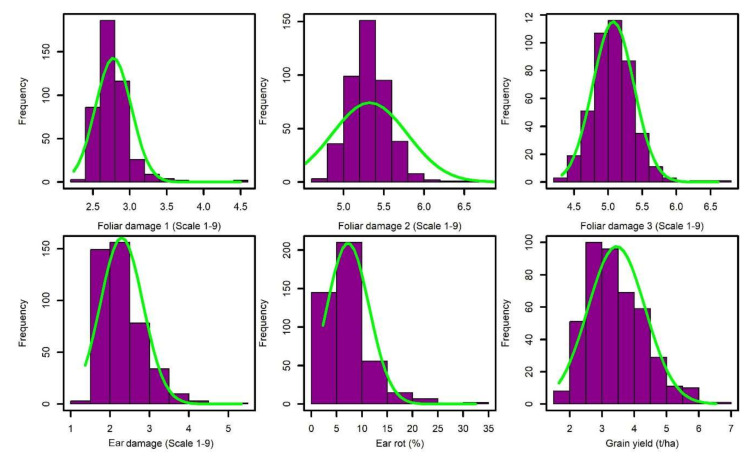
Frequency distribution of means for foliar damage scores 7, 14, and 21 days after artificial infestation (foliar damage score 1, 2, and 3), ear damage, ear rot (%), and grain yield (t/ha).

The frequency distribution of the BLUEs for foliar damage scores at 7, 14, and 21 days after artificial infestation, ear damage, ear rot, and grain yield exhibited approximately near normal distributions (Figure 1). Evaluation of the frequency distributions of the phenotype data indicated that further parametric tests could therefore be implemented without violating the model assumptions of normality. The best performing lines under the artificial infestation of FAW over two seasons are listed in Table 1. Leaf damage scores 7, 14, and 21 days after artificial FAW infestation averaged 2.8, 5.3, and 5.1, respectively (Table 2). Transgressive segregants were observed in both directions of the injury rating scale. Most lines recorded high injury ratings and were considered as susceptible to FAW leaf feeding damage, while a few lines exhibited injury ratings that were not significantly different from those of the resistant CML71 control. These lines were earmarked as good sources of native resistance to FAW foliar feeding damage. Injury ratings for lines identified as FAW-susceptible were not significantly different to those of the susceptible CML444 control. Most lines did not suffer severe ear damage on a scale of 1–9 and therefore the observed ear damage mean was low (2.3). Ear rot averaged 7.3% while grain yield under artificial FAW infestation had a mean of 3.4 tons/ha with a range from 1.6 to 6.5 tons/ha.

**Figure 2:**
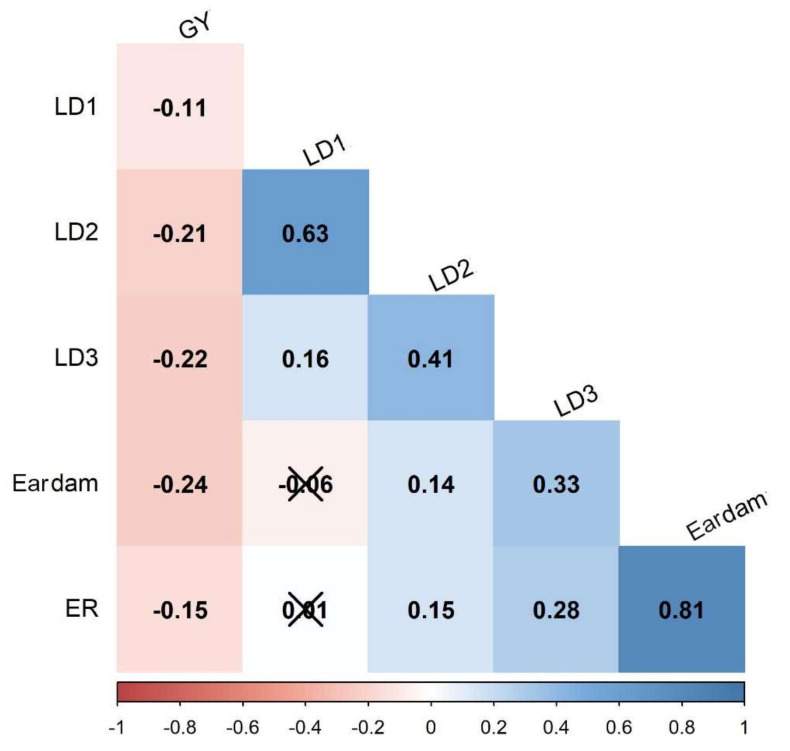
Pairwise Pearson correlation analysis for six traits associated with FAW resistance. LD1, LD2, LD3: foliar damage rating 7, 14, and 21 days after artificial FAW infestation, respectively; Eardam—ear damage, ER—ear rot, GY—grain yield.

Knowledge on the proportion of genetic variation for the traits of interest is critical to a breeder when formulating an efficient and effective resistance breeding scheme. Analysis of variance (ANOVA) was used to partition total variance into genotypic, phenotypic, and variance due to genotype by environment interaction (Table 1). Across-environment analyses revealed significant (*p* < 0.01) genotypic and genotype by environment interaction variances for all traits. Heritability (h^2^) estimates for foliar damage scores were moderate with 0.58, 0.45, and 0.38 at 7, 14, and 21 days after artificial FAW infestation, respectively. Grain yield recorded the highest (0.62) heritability estimate when compared to the rest of the traits. Ear damage, ear rot, and grain yield showed moderately high coefficients of variation estimates, which suggested that the selection of FAW-resistant lines based on these traits could be effective. Pairwise Pearson correlation analysis revealed significant but negative correlations between grain yield and the rest of the traits (Figure 2). Foliar damage scores at 7 days after artificial infestation showed no significant correlation with ear rot and ear damage. The results showed that ear damage was significant and positively correlated (0.81) with ear rot.

### 3.2. PCA, Kinship and LD Analysis

**Figure 3:**
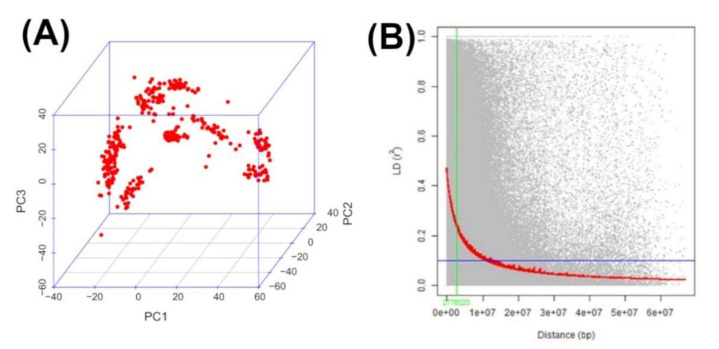
Population structure analyses of GWAS panel with first three principal components plot (**A**) based on the observed genotype data and genome wide linkage disequilibrium (LD) plot (**B**). Horizontal blue line in LD plot represents the 95th percentile of the distribution of unlinked r^2^ while the inner fitted trend line is the non-linear logarithmic regression curve of r^2^ on genetic distance.

GWAS statistical power and resolution is reduced if the phenotypes of interest are significantly correlated with relatedness or population structure [57]. PCA based on the observed genotype data showed no clear pattern of population stratification (Figure 3A). The proportion of total variability explained by the first, second, and third principal components (PC1–3) was 9%, 6%, and 5%, respectively. Figure 3A shows a three-dimensional scatter plot of the first three principal components which together contributed about 20% of the total variation in the data.

Figure 3B shows the extent of the genome-wide distribution of LD. The y-axis values represent the squared correlation coefficient (r^2^) between pairs of SNPs while the x-axis values represent the physical distance in Mega base pairs (Mbp). The red line is the moving average of the 10 adjacent markers. Each dot represents a pair of distances between two markers on the window and their squared correlation coefficient. Across the ten chromosomes, the magnitude of r^2^ dropped sharply as the genetic distance between SNP markers increased. At LD cut off points of r^2^ = 0.1 (threshold value beyond which LD was likely to be caused by linkage), the average physical distance was 10 Mbp.

### 3.3. Genome-Wide Association Analysis

**Figure 4:**
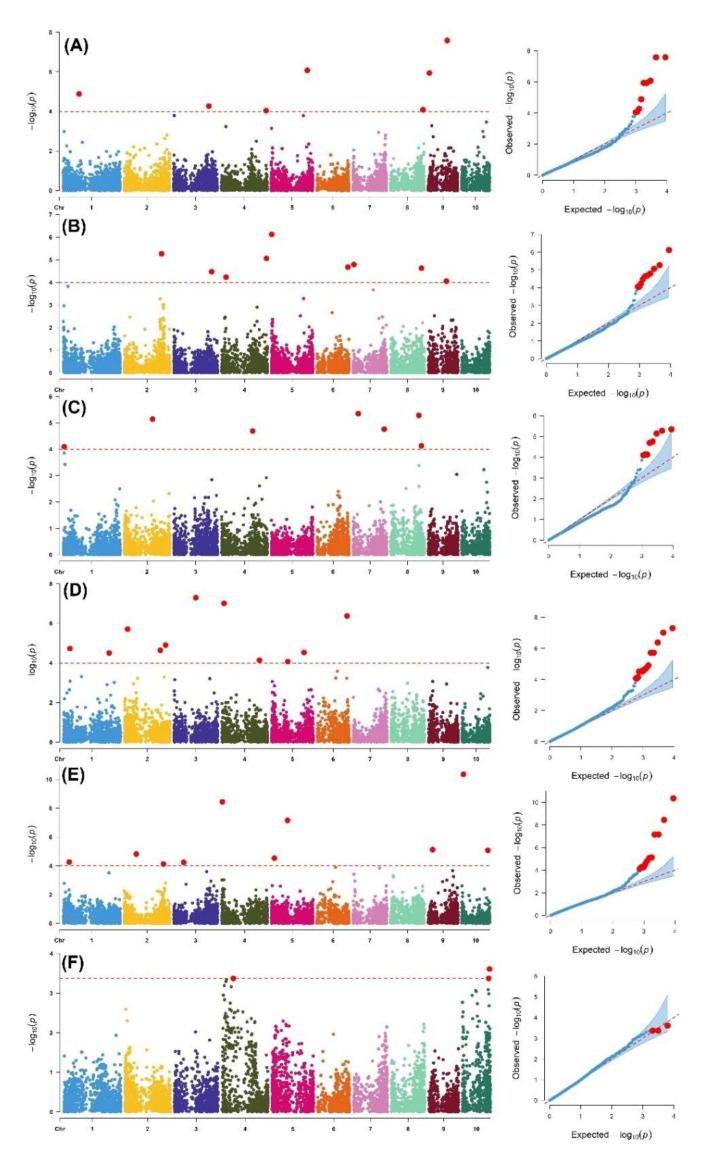
Manhattan plots and associated Q–Q plots derived from the GWAS analysis of six traits: (**A**) leaf damage score 1, (**B**) leaf damage score 2, (**C**) leaf damage score 3, (**D**) ear damage, (**E**) ear rot, and (**F**) grain yield. On the Manhattan plots, the x-axis shows SNP locations along the 10 chromosomes while the y-axis shows the level of statistical significance as measured by the negative log of the corresponding *p*-value for each SNP. Genome-wide significance level for marker-trait associations was plotted as the horizontal dotted line on the Manhattan plots. The y-axis on the Q–Q plots represents the observed association *p*-values while the x-axis represents the expected uniform distribution of *p* values.

Results from the genome-wide association analysis of the six traits relevant to FAW resistance are presented in Manhattan plots (Figure 4). Diagnostic Q–Q plots displayed beside each Manhattan plot were used to assess the suitability of the GWAS model used. A set of nine SNPs located on chromosomes 1, 3, 4, 5, 8, and 9 were found to be most significantly associated with foliar damage scores 7 days after the artificial infestation of FAW while 11 SNPs distributed across all chromosomes except chromosome 10 were significantly associated with foliar damage scored on the 14th day after infestation (Figure 4, Table 3). A total of eight SNPs distributed on chromosomes 1, 2, 4, 7, and 8 were highly associated with the foliar damage score on the 21st day after artificial FAW infestation. Two SNPs (DT9_102187311 and DT5_193883551) with the strongest association (*p* = 10–8) with foliar damage scores across all infestation durations were found. For ear damage scores, 13 SNPs distributed on chromosomes 1, 2, 3, 4, 5, 6, and 10 were detected. There were 13 SNPs significantly associated with ear rot resistance distributed across all chromosomes except chromosome 8. SNP DT5_86480332 was commonly detected for both ear damage and ear rot scores. Grain yield under artificial FAW infestation exhibited large peaks of association signals on chromosomes 4 and 10 (Figure 4F, Table 3). There were no common SNPs detected between different foliar damage scores, ear damage, and grain yield data.

The magnitude of the genetic effects of each SNP were determined from the final GWAS model and the signs of the genetic effect were used to identify the allele influencing the trait. The major allele increased the expression of the trait while the minor allele reduced the trait expression. The SNP (*DT8_165270110*) located on chromosome 8 contributed the strongest estimated effect size (6.50) for the expression of the leaf feeding damage resistance trait. A set of putative candidate genes associated with significant SNPs were identified and their inferred biological functions varied from a defense response to a carbohydrate metabolic process (Table 3).

**Figure 5:**
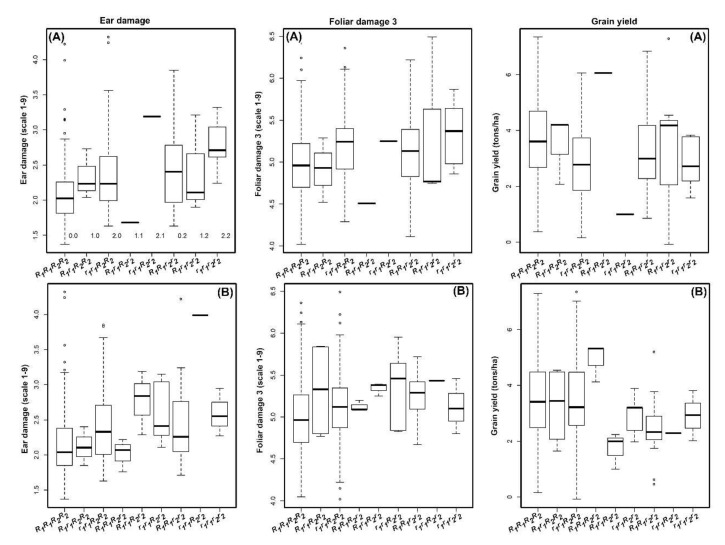
Box plots showing the phenotypic values of the different allele classes with combination of two SNPs in boxplots A with *DT1_34838367* (CC/TT = R_1_R_1_/r_1_r_1_) and *DT2_192225273* (AA/CC -R_2_R_2_/r_2_r_2_) and boxplots B with *DT5_86480332* (AA/TT = R_1_R_1_/r_1_r_1_) and *DT6_108383751* (AA/CC = R_2_R_2_/r_2_r_2_). R for resistance and r is for susceptible to FAW. These SNPs were identified for ear damage score and showed their effect also on foliar damage and grain yield under FAW infestation. The black horizontal lines in the middle of the boxes are the median values for the trait performance in the respective allele classes.

The phenotypic values of the different allele classes of these SNPs in the association panel for ear damage, foliar damage, and grain yield under FAW infestation are presented in Figure 5. Among several genomic regions identified for ear damage scores, allelic effects on FAW resistance were prominent in four selected SNPs (*DT1_34838367* (CC/TT) and *DT2_192225273* (AA/CC) and boxplots B with *DT5_86480332*(AA/TT) and *DT6_108383751*). Two SNPs *DT1_34838367* (CC/TT) and *DT2_192225273* (AA/CC) in a combination of favorable alleles resulted in the score of 2.0 and 4.9 for ear damage and foliar damage, respectively (Figure 5A). On the contrary, for the same marker with unfavorable alleles, the scores were increased to 2.8 and 5.5, respectively. Similarly, for the other two SNPs *DT5_86480332* (AA/TT) and *DT6_108383751*, a decrease in ear damage score and foliar damage score and an increase in grain yield were also observed (Figure 5B).

**Figure 6:**
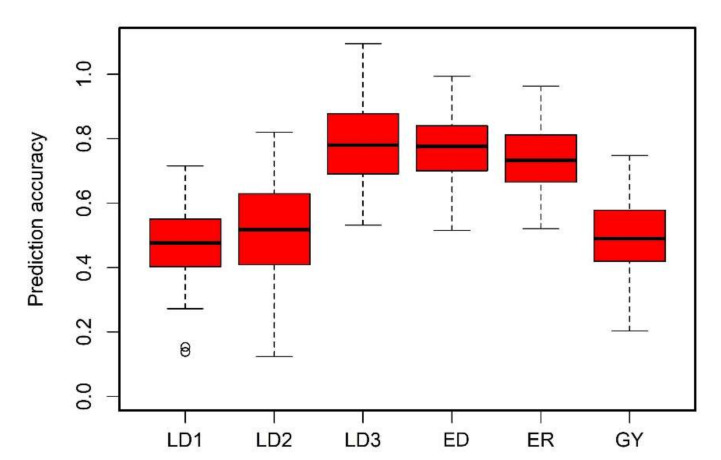
Box plots showing genomic prediction accuracy for six traits evaluated under artificial FAW infestations. LD1, LD2, LD3: foliar damage rating at 7, 14, and 21 days after artificial FAW infestation, respectively; ED—ear damage, ER—ear rot, GY—grain yield.

The RR-BLUP model was used to estimate the performance of maize genotypes for FAW resistance-associated traits (Figure 6). Average prediction accuracies across the studied genotypes were higher for the foliar damage score on the 21st day after the artificial infestation of FAW (0.79) and lower for foliar damage on the 7th day after the artificial infestation of FAW (0.48). In the GWAS panel, we observed the prediction accuracy of 0.48, 0.52. 0.79, 0.77, 0.73, and 0.50 for foliar damage on the 7th day, 14th day, and 21st day, ear damage, ear rot, and grain yield under FAW infestation, respectively.

## 4. Discussion

Inadequate maize production, particularly due to insect-pest infestation, does not only threaten the livelihoods of millions of smallholder farmers who dominate maize agriculture in Africa, but also undermines the hunger reduction plan envisioned in the 2030 sustainable development goals [58]. The need to sustainably protect maize crop from FAW foliar damage and to mitigate subsequent yield losses using a multi-pronged strategy that includes the utilization of maize varieties endowed with native resistance is a prioritized research agenda in Africa [59]. The objectives of the present study were to screen maize lines developed by CIMMYT for their reactions to artificial FAW infestation, identify FAW resistant germplasm for use in future resistance breeding, and investigate the genetic basis of the resistance trait.

A comparison of injury ratings 7, 14, and 21 days after artificial infestation showed intensive FAW foliar feeding during the early to mid-whorl vegetative stage of the maize plant growth and development. This is because FAW feeds intensively during its 5th and 6th growth stages. The most destructive FAW developmental stage may also coincide with the most susceptible maize growth stage. Maize lines that exhibited injury ratings that were not significantly different from those of the resistant control (Table 1) could be utilized in developing novel FAW-resistant populations.

Grain yield, the most important agronomic trait, was low under artificial FAW infestation. This could also be due to the extensive foliar damage which causes a direct loss of photosynthates and alteration of the normal functioning of the remaining leaf tissue [60]. Foliar feeding by FAW reduces maize leaf surface area, which negatively affects the photosynthesis process and assimilate partitioning, both of which are critical in grain filling. Stem tunnelling caused by FAW also disrupts water and nutrients uptake, which could adversely impact grain yield. Estimates of genetic variation among traits of interest provide useful information when formulating a resistance breeding plan. For all traits, phenotypic variances were higher than the corresponding genotypic variances, which suggested the considerable influence of environmental factors as well as genotype by environment interaction on the expression of these traits.

The potential of phenotypic plasticity to evolve [61] in maize lines endowed with native resistance to FAW foliar damage due to the strong influence of genotype by environment (GXE) interaction underscores the importance of conducting multi-year germplasm evaluations when screening maize germplasm for resistance to FAW foliar damage. Previous studies have also concluded that the analysis of genotype by environment interactions could facilitate the identification of cultivars whose yield-stability are related to the linear effect of an environmental index [62]. The moderately high component of heritable variation associated with foliar damage scores 7 and 14 days after artificial infestation indicated that these traits are amenable for improvement. Leaf damage scores 21 days after artificial infestation had the lowest heritability estimate, which suggested the precise evaluation of early-stage foliar damage compared to late-stage foliar damage. Heritability estimates reported in the present work could help optimize the choice of the most progressive breeding method for use in trait improvement. The missing part of heritability [63], however, can be uncovered by utilization of new genetic study designs that incorporate more novel types of genotypes to unravel rare alleles of large effect [64]. Although grain yield under artificial FAW infestation exhibited the highest heritability estimate (0.62), a direct selection of a FAW resistance germplasm based on grain yield component could be ineffective. This is because grain yield is also a complex trait influenced by the interaction of various yield components such as plant height, ear height, and 1000 grain weight [65]. High coefficients of variation for all traits suggested that the selection of FAW-resistant lines based on these traits could be effective.

The magnitude and the direction of the correlations for several FAW resistance indicator traits should be examined to understand the desirability of their relationships with grain yield, which is the most desirable agronomic trait with economic importance. Results from the correlation analysis indicated that grain yield was negatively correlated with a high magnitude to foliar damage scores, ear rot, and ear damage traits. The correlation between ear rot and ear damage was positive, strong, and highly significant (*p* < 0.01). FAW-damaged ears are predisposed to fungal attacks, rots, and mycotoxin contamination which adversely impact grain quantity and quality [8]. Interestingly, ear damage was not significantly correlated with the early stage of the foliar damage score but was significantly and positively correlated with the foliar damage score recorded at later stages (Figure 2).

In the present study, SNPs considered for GWAS had a fairly even marker distribution spanning the whole maize genome (Supplementary Figure S1). The uneven distribution of markers may contribute to the detection of false positives and biased estimation of population structure and relatedness [66]. In the GWAS association test model implemented, population structure and kinship in the association mapping panel were integrated as covariates to reduce the detection of spurious associations. The assessment of the diagnostic Q–Q plots indicated that population structure and kinship were effectively controlled. PCA and kinship analysis (Supplementary Figure S2) suggested that there was a substantial amount of genetic differentiation in the association mapping panel and weak evidence to explain the presence of a population structure.

The rate of linkage disequilibrium decay provides useful information required to implement meaningful association mapping study [67]. In the present study, LD persisted over a large genetic distance (r^2^ < 0.1 within 10 Mega base pairs). The high level of homozygosity in the maize lines used in the current study may have rendered recombination ineffective in breaking down LD. Varying estimates of LD decay have been reported in maize, such as 27.31 kb [68] and 14.97 kb in an IMAS panel [69]. These studies indicated a rapid decay in the tropical maize germplasm as compared to the temperate germplasm, which suggests a broader genetic base, resulting from high recombination events [70]. This provides breeders with an opportunity to select germplasm that integrates high grain yield with FAW resistance, disease resistance, and abiotic stress tolerance.

GWAS revealed 56 significant marker–trait associations (Table 3). Chromosome 4 accounted for the highest number (15%) of the SNP markers associated with foliar damage. Chromosomes 4 and 9 have been reported to harbor SNP markers associated with resistance to major lepidopteran pests in maize [71]. One major effect QTL on chromosome 9 in bin 9.03, reported in previous studies [72,73], coincided with SNP DT9_96875821, detected for the foliar damage score on the 14th day after infestation. Another major QTL detected on bin 4.06 coincided with SNP DT4_167218393, detected for the foliar damage score 21 days after infestation.

The shift in allele frequency proportions has been attributed to the natural or artificial selection of variants conferring a selective advantage [74]. The results from this study suggest that the frequency of alleles conferring an increased expression of resistance to FAW foliar damage are rare and could increase over generations to become fixed or common in maize populations due to selection. In the present study, SNP loci with high minor allele frequencies had small effects, while those with low allele frequencies had larger effect sizes. Whereas the effect sizes of individual loci were small, their potential to confer a durable and stable resistance to FAW feeding damage depends on their combined effect sizes [64]. Our results corroborate with previous findings that have linked the genetic basis of pest resistance in maize to multiple genes of small effects that are scattered across the genome [29].

Larger peaks on the Manhattan plots were suggestive of a strong association between the surrounding genomic region and the corresponding phenotypic trait and warrants further validation. Grain yield under artificial FAW infestation, for instance, exhibited peaks of association signals on chromosomes 4 and 10 which could be due to strong selection pressure during the breeding and domestication process [75]. While biological functions of uncharacterized candidate genes could not be inferred, SNP (*DT8_165429441*), strongly associated (*p* = 10^−5^) with leaf damage scores 14 days after infestation, was found within a genomic region containing the GRMZM2G016802 gene that participates in the defense response by restricting injury occurrence and enhancing recovery after injury [23]. The allelic effect of some of the selected markers (two SNPs in boxplots A with *DT1_34838367* and *DT2_192225273* and boxplots B with *DT5_86480332* and *DT6_108383751*) clearly support the effective role in improving the level of FAW resistance (Figure 5). These consistent regions or SNPs could potentially help the breeders to design an effective strategy to introgress these QTNs in relevant breeding materials through marker-assisted breeding.

Foliar damage in maize triggers a complex cascade of biological pathways mediated by various genetic factors, effector molecules, and signaling components and which leads to the accumulation of secondary metabolites with anti-feedant effects [76]. SNP (DT3_3627288) located in chromosome 3 and significantly associated with late-stage foliar damage scores, for instance, was mapped in a genomic region adjacent to the GRMZM2G045259 gene involved in the phosphorylation–dephosphorylation of proteins in a process catalyzed serine/threonine kinases to effect signal transductions that play a prominent role in plant defense mechanisms [77]. The preferential upregulation of genes was implicated in general stress responses such as receptor kinases and in plants colonized by foliar feeding insects [78]. SNP (DT1_26003816), significantly associated with the ear damage trait, suggested that the putative candidate genes GRMZM2G015804 are involved in the carbohydrate metabolic process. In plants under biotic attack [79], photosynthetic genes involved in metabolic processes have been reported to be downregulated, and this could explain low grain yield under FAW infestation. Compared to plant–pathogen resistance genes, little information exists on plan–-insect resistance genes. To fully elucidate the genetic basis of FAW resistance in maize, further investigations are therefore warranted to functionally validate putative candidate genes with unknown functions reported in this study.

GS facilitates the rapid selection of superior genotypes through ease in genotyping, which captures the maximum favorable alleles. The potential of GS models in identifying lines with favorable alleles in maize for different traits has been studied by different groups [25,69,80]. The moderate to high accuracies observed in this study (Figure 6) for the association panel offer promise in breeding for FAW resistance. The prediction accuracy of the association panel was in agreement with various studies on moderately complex traits such as striga [81], maize chlorotic mottle virus [82], MLN [83], and grey leaf spot [69]. Significant genetic structure and a high LD between adjacent markers of the diversity panel result in a moderate prediction accuracy, which could also be attributed to its moderate heritability [82]. The rapid decline in the cost of genotyping makes it possible to routinely apply GS in breeding, specifically for complex traits such as FAW resistance. Combining GWAS and the predictive capabilities of GS will also improve the prediction accuracy by using information on the major QTLs detected in GWAS. Overall, the predicted accuracies are moderate, and under the assumption of the three cycles per year possibility, a high selection gain for complex traits such as FAW resistance is achievable with optimal resources.

## 5. Conclusions

To investigate the genetic basis of FAW resistance, we employed a single panel consisting of 423 tropical maize lines for GWAS and genomic prediction. The phenotypic correlations of the FAW resistance traits investigated indicated that this panel can be used to select better-performing lines under FAW infestation. GWAS identified 56 SNPs associated with FAW resistance traits. The genomic regions identified can be used for selection efforts to enhance FAW resistance. Furthermore, the findings showed that including GS in maize breeding can successfully support phenotypic selection to improve maize native genetic resistance. Future work should, therefore, focus on validating the identified SNPs to enhance the efficacy of maize breeding in SSA.

## Figures and Tables

**Table 1:** Best performing 15 lines evaluated under artificial infestation of FAW in net houses for two environments.

Genotype	LD1	LD2	LD3	Eardam	ER (%)	GY (tons/ha)
*CKIR04005*	2.85	4.83	4.82	1.98	3.16	6.54
*CKDHL1920804*	2.93	5.41	4.94	2.09	4.14	6.31
*CKDHL1922286*	2.64	5.09	4.94	2.11	11.18	5.99
*CKDHL1920877*	2.33	4.83	4.45	2.16	5.96	5.95
*CKDHL1923759*	3.04	5.56	4.74	1.92	3.42	5.84
*CKDHL1921755*	2.62	5.48	5.49	2.26	9.67	5.80
*CKDHL1922245*	2.62	5.06	4.89	2.03	5.55	5.63
*CKDHL1922428*	2.60	5.43	5.02	2.26	5.72	5.57
*CKDHL1922266*	2.87	5.24	4.98	2.03	5.87	5.57
*CKDHL1922467*	2.66	5.11	5.22	2.68	13.53	5.57
*CKDHL1920715*	2.47	4.93	4.82	2.01	4.34	5.54
*CKDHL1920580*	3.08	5.51	5.11	2.21	8.32	5.54
*CKDHL1924395*	2.77	5.39	4.96	2.93	15.31	5.45
*CKDHL1922258*	3.43	5.81	4.53	2.36	5.86	5.44
*CKDHL1924391*	2.61	5.27	5.09	2.28	5.15	5.40

LD1, LD2, LD3 = foliar damage rating at 7, 14, and 21 days after artificial FAW infestation, respectively; Eardam = ear damage, ER= ear rot, GY= grain yield.

**Table 2:** Genetic parameters for FAW GWAS panel evaluated under artificial infestation of FAW in net houses for two environments.

Trait	Mean	σ^2^_G_	σ^2^_GxE_	σ^2^_e_	h^2^	LSD_5%_	CV (%)
LD1	2.78	0.06 **	0.04 **	0.10	0.58	0.42	7.71
LD2	5.32	0.09 **	0.05 **	0.35	0.45	0.65	6.21
LD3	5.07	0.08 *	0.18 **	0.18	0.38	0.59	5.91
Eardam	2.28	0.14 **	0.14 **	0.19	0.55	0.62	13.82
ER	7.26	14.78 **	16.19 **	21.97	0.52	6.63	46.44
GY	3.45	0.97 **	0.42 **	1.54	0.62	1.74	25.69

**σ^2^_G_** = Genotypic variance; **σ^2^_e_** = error variance; **σ^2^_GxE_** = genotype by environment interaction; h^2^ = broad-sense heritability; LSD = least square difference; CV = coefficient of variation; LD1, LD2, LD3 = foliar damage rating at 7,14, and 21 days after artificial FAW infestation, respectively; Eardam = ear damage, ER= ear rot, GY= grain yield. *, ** Significant at *p* < 0.05 and *p* < 0.01 level, respectively.

**Table 3:** Significantly associated single-nucleotide polymorphisms (SNPs) along with the predicted gene model and their function detected by genome-wide association studies in CIMMYT association mapping panel for FAW resistance-associated traits.

SNP	Chr	MLM-*p* Value	MAF	Allele Effect	Putative Candidate Genes	Predicted Function of Candidate Gene
Foliar damage 1						
DT1_86151758	1	1.32 × 10^−05^	0.34	−0.04	No hit	-
DT3_189787778	3	5.43 × 10^−05^	0.17	−0.06	GRMZM2G024992	Uncharacterized
DT3_3627388	3	1.63 × 10^−04^	0.31	0.05	GRMZM2G045259	ATP binding, protein serine threonine kinase activity
DT4_240657423	4	9.17 × 10^−05^	0.17	0.06	GRMZM2G124151	Transferring glycosyl groups
DT5_193883551	5	8.48 × 10^−07^	0.11	0.08	No hit	-
DT5_172818477	5	1.67 × 10^−04^	0.40	−0.04	GRMZM2G038536	Base excision repair (BER) pathway, by catalyzing the ADP-ribosylation of acceptor proteins involved in chromatin architecture and DNA metabolism.
DT8_174365183	8	8.22 × 10^−05^	0.46	−0.05	GRMZM2G370044	Uncharacterized
DT9_102187311	9	2.62 × 10^−08^	0.44	−0.06	GRMZMG036921	Transfer RNA intron lyase
DT9_6789620	9	1.16 × 10^−06^	0.39	−0.02	GRMZM2G017257	Chloroplast accumulation movement, ATP-dependent microtubule motor activity
Foliar damage 2						
DT1_26003816	1	1.49 × 10^−04^	0.43	0.04	GRMZM2G015804	Carbohydrate metabolic process
DT2_200197508	2	5.45 × 10^−06^	0.49	0.04	GRMZM2G703307	Integral component of membrane
DT3_203549581	3	3.39 × 10^−05^	0.14	−0.06	GRMZM2G078756	Phenylalanyl-tRNA aminoacylation
DT4_241323024	4	8.75 × 10^−06^	0.48	0.05	GRMZM2G051004	NAD binding
DT4_24639576	4	5.93 × 10^−05^	0.39	−0.04	GRMZM2G088169	Cell fate determination
DT5_1996596	5	7.65 × 10^−07^	0.45	−0.05	GRMZM2G415498	DNA-mediated transposition,
DT6_166058896	6	2.12 × 10^−05^	0.27	−0.04	GRMZM2G094892	Regulation of long-day photoperiodism, flowering
DT7_4773701	7	1.63 × 10^−05^	0.39	0.04	GRMZM2G480002	Uncharacterized
DT7_108615586	7	2.16 × 10^−04^	0.20	0.06	No hit	-
DT8_165429441	8	2.36 × 10^−05^	0.21	0.06	GRMZM2G016802	Defense response
DT9_96875821	9	8.88 × 10^−05^	0.40	0.04	GRMZM2GO48919	Uncharacterized
Foliar damage 3						
DT1_5722917	1	8.05 × 10^−05^	0.30	0.07	GRMZM2G319022	Uncharacterized
DT1_6086007	1	1.40 × 10^−04^	0.29	−0.05	GRMZM2G341918	Uncharacterized
DT2_151852785	2	7.22 × 10^−06^	0.43	0.06	No hit	-
DT4_167218393	4	2.04 × 10^−05^	0.34	−0.07	GRMZM2G168369	Uncharacterized
DT7_27787652	7	4.50 × 10^−06^	0.40	0.06	GRMZM2G097719	Uncharacterized
DT7_167536749	7	1.75 × 10^−05^	0.23	−0.08	GRMZM2G017145	Protein dimerization activity
DT8_151149212	8	5.24 × 10^−06^	0.39	−0.07	GRMZM2G043117	Hydrotropism
DT8_165270110	8	7.42 × 10^−05^	0.32	6.50	GRMZM2G114046	Cellular macromolecule, metabolic process, integral component of membrane
Ear damage						
DT1_34838367	1	1.90 × 10^−05^	0.36	−0.12	No hit	-
DT1_245864468	1	3.17 × 10^−05^	0.49	−0.12	No hit	-
DT2_16546600	2	1.95 × 10^−06^	0.45	0.00	GRMZM2G042756	Dehydration responsive element binding protein, DNA-binding transcription factor activity
DT2_220742831	2	1.27 × 10^−05^	0.28	−0.13	GRMZM2G077256	Uncharacterized
DT2_192225273	2	2.31 × 10^−05^	0.10	−0.14	No hit	-
DT3_117394631	3	5.07 × 10^−08^	0.10	0.20	No hit	-
DT4_12954089	4	9.87× 10^−08^	0.34	0.16	GRMZM2G377115	Chlorophyll catabolic process, response to water deprivation
DT4_202434317	4	7.31 × 10^−05^	0.41	−0.08	No hit	-
DT5_174018428	5	2.99 × 10^−05^	0.27	0.12	No hit	-
DT5_86480332	5	8.55 × 10^−05^	0.22	−0.09	No hit	-
DT6_159770879	6	4.30 × 10^−07^	0.11	0.12	No hit	-
DT6_108383751	6	2.65 × 10^−04^	0.44	0.11	GRMZM5G809695	Regulation of cell growth
DT10_138678949	10	1.71 × 10^−04^	0.18	−0.09	No hit	-
Ear rot						
DT1_32439894	1	5.56 × 10^−05^	0.08	0.15	No hit	-
DT2_64332803	2	1.53 × 10^−05^	0.45	−0.87	GRMZM2G373828	
DT2_210205836	2	7.53 × 10^−05^	0.19	−1.06	GRMZM2G102138	
DT3_53768832	3	5.82 × 10^−05^	0.20	−0.65	No hit	-
DT4_4804227	4	3.62 × 10^−09^	0.33	1.00	AC214255.3_FG008	
DT5_86480332	5	7.01 × 10^−08^	0.22	1.37	No hit	-
DT5_15869219	5	2.93 × 10^−05^	0.15	1.10	No hit	-
DT6_99748720	6	1.26 × 10^−04^	0.17	−0.75	AC215906.3_FG001	
DT7_142857000	7	1.46 × 10^−04^	0.22	0.83	GRMZM2G074472	
DT9_23210980	9	7.53 × 10^−06^	0.36	0.63	No hit	-
DT9_130788433	9	2.17 × 10^−04^	0.47	0.39	GRMZM2G043295	UDP-glycosyltransferase activity
DT10_8711707	10	4.37 × 10^−11^	0.15	−1.76	GRMZM2G167999	Transducin/WD40 repeat-like superfamily protein
DT10_140174620	10	8.46 × 10^−06^	0.20	1.01	GRMZM5G887345	PF00179: Ubiquitin-conjugating enzyme
Grain yield						
DT4_60154355	4	4.28 × 10^−04^	0.23	−0.26	No hit	-
DT10_145757365	10	2.46 × 10^−04^	0.40	0.25	GRMZM2G0101264	Uncharacterized
DT10_141438482	10	4.28 × 10^−04^	0.42	−0.30	No hit	-

SNP = Single nucleotide polymorphism, Chr = chromosome, MAF = minor allele frequency, *p*-value = adjusted *p*-values following a false discovery rate control procedure, Effect = allelic effect estimates per SNP. Code names for markers, e.g., DT4_60154355 indicates a marker in chromosome 4 at position 60154355 base pairs (using B73 maize reference genome version 3); (-) = unknown.

## Data Availability

All datasets generated for this study are included in the article. The DART marker data are available at https://data.cimmyt.org.

## Supplementary Materials

The following are available online at https://www.mdpi.com/article/10.3390/genes13020251/s1, Figure S1: Density plot showing the number SNPs within 1 Mb window size from the studied 7100 markers. The number of SNPs is displayed on a scale from green to red; Figure S2: Kinship analysis of the FAW GWAS panel. The heat map shows the pairwise kinship matrix based on 7100 filtered SNPs.
