# Supplementary material for: Genomic Analysis of Resistance to Fall Armyworm (Spodoptera frugiperda) in CIMMYT Maize Lines
Source: Genes (Basel). 2022 Jan 28;13(2):251. doi: 10.3390/genes13020251 (PMC8872412; doi:10.3390/genes13020251)
Supplement: Supplementary file 1 [file genes-13-00251-s001.zip › genes-1533498-supplementary.pdf]

Supplementary Materials

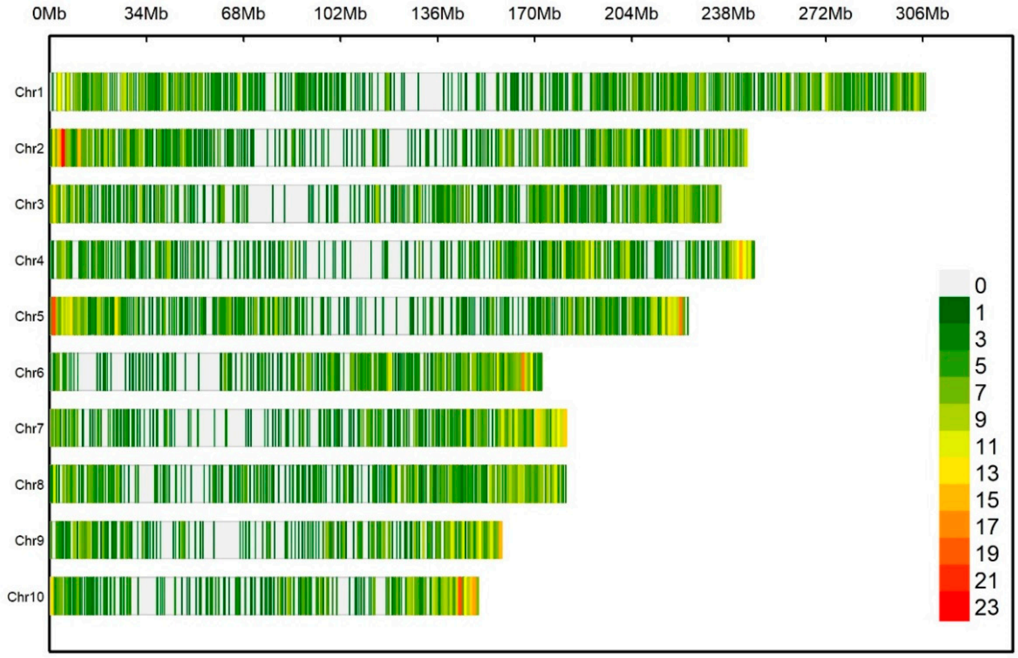

Supplementary Figure S1. Density plot showing the number SNPs within 1 Mb window size from the studied 7,100 markers. The number of SNPs is displayed on a scale from green to red.

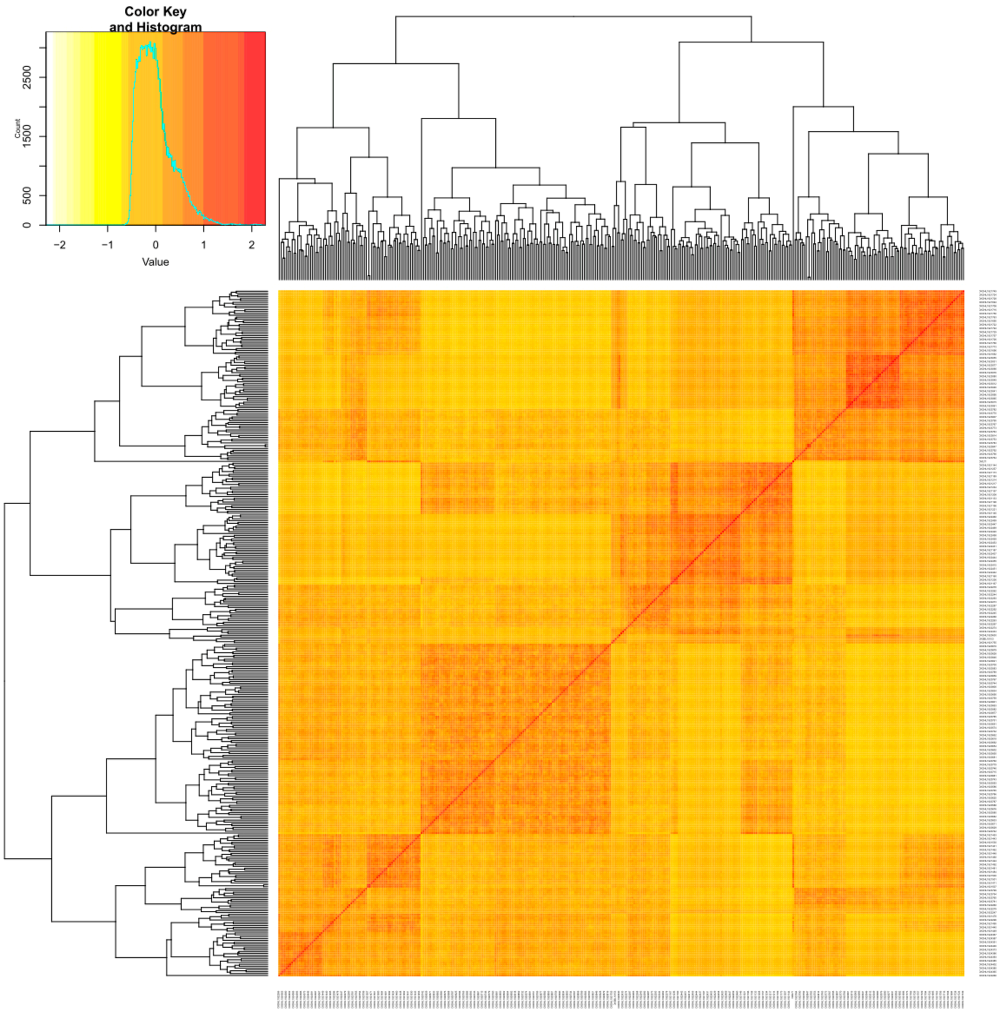

Supplementary Figure S2. Kinship Analysis of the FAW GWAS panel. The heat map shows the pairwise kinship matrix based on 7,100 filtered SNPs.
